# Adrenocortical tumor in patients with congenital adrenal hyperplasia due to 21-hydroxylase deficiency

**DOI:** 10.1186/1687-9856-2015-S1-P51

**Published:** 2015-04-28

**Authors:** Vu Chi Dung, Bui Phuong Thao, Nguyen Phu Dat, Nguyen Thi Hoan, Tran Van Khanh, Ta Thanh Van

**Affiliations:** 1 National Hospital of Pediatrics, Hanoi, Vietnam; 2 Hanoi Medical University, Hanoi, Vietnam

## 

Adrenocortical tumour have been described in patients with 21-hydroxylase deficiency. These tumours are usually considered to be ACTH – dependent, as diffuse adrenal cortical hyperplasia is commonly seen. We report adrenal cortical tissue tumours developed in three patients with untreated congenital adrenal hyperplasia due to 21-hydroxylase deficiency. All of them had symptoms of adrenogenital vililizing syndrome. A diagnosis of adrenocortical tumour was established by the symptoms, hormonal profile, ultrasonography, and adrenal CT scan. Two of the tumours were located in the right side, all the patients were performed surgery before hormonal replacement therapy because of evidence of secreated adrenal tumors, and the histological diagnosis indicated an adrenocortical adenoma. After removal of tumours, the size of adrenal gland was monitored by serial ultrasonography, and the congenital adrenal hyperplasia was confirmed by extremely high levels of basal serum testosterone, 17-OHP levels, increasing virilizing syndrome after surgery, diffuse hyperplasia of adrenal gland and identified mutations in CYP21A2 gene.

